# The Ectodomain of Glycoprotein from the Candid#1 Vaccine Strain of Junin Virus Rendered Machupo Virus Partially Attenuated in Mice Lacking IFN-αβ/γ Receptor

**DOI:** 10.1371/journal.pntd.0004969

**Published:** 2016-08-31

**Authors:** Takaaki Koma, Cheng Huang, Judith F. Aronson, Aida G. Walker, Milagros Miller, Jeanon N. Smith, Michael Patterson, Slobodan Paessler

**Affiliations:** Department of Pathology, University of Texas Medical Branch at Galveston, Galveston, Texas, United States of America; Colorado State University, UNITED STATES

## Abstract

Machupo virus (MACV), a New World arenavirus, is the etiological agent of Bolivian hemorrhagic fever (BHF). Junin virus (JUNV), a close relative, causes Argentine hemorrhagic fever (AHF). Previously, we reported that a recombinant, chimeric MACV (rMACV/Cd#1-GPC) expressing glycoprotein from the Candid#1 (Cd#1) vaccine strain of JUNV is completely attenuated in a murine model and protects animals from lethal challenge with MACV. A rMACV with a single F438I substitution in the transmembrane domain (TMD) of GPC, which is equivalent to the F427I attenuating mutation in Cd#1 GPC, was attenuated in a murine model but genetically unstable. In addition, the TMD mutation alone was not sufficient to fully attenuate JUNV, indicating that other domains of the GPC may also contribute to the attenuation. To investigate the requirement of different domains of Cd#1 GPC for successful attenuation of MACV, we rescued several rMACVs expressing the ectodomain of GPC from Cd#1 either alone (MCg1), along with the TMD F438I substitution (MCg2), or with the TMD of Cd#1 (MCg3). All rMACVs exhibited similar growth curves in cultured cells. In mice, the MCg1 displayed significant reduction in lethality as compared with rMACV. The MCg1 was detected in brains and spleens of MCg1-infected mice and the infection was associated with tissue inflammation. On the other hand, all animals survived MCg2 and MCg3 infection without detectable levels of virus in various organs while producing neutralizing antibody against Cd#1. Overall our data suggest the indispensable role of each GPC domain in the full attenuation and immunogenicity of rMACV/Cd#1 GPC.

## Author Summary

Machupo virus (MACV), a member of *Arenaviridae* family, causes Bolivian hemorrhagic fever (BHF) in humans. No approved vaccine or treatment are available to date despite the high case fatality rate of BHF. rMACV/Cd#1-GPC is fully attenuated and protects mice from lethal MACV challenge. Although one virulence determinant was found in the transmembrane domain of GPC (F438), other virulence determinants in GPC are very likely. Our new data indicated that the ectodomain of Cd#1 GPC is necessary, but not sufficient, for complete attenuation of the rMACV/Cd#1-GPC. This new finding may help generate highly attenuated MACVs for vaccine development and/or for drug screening purpose.

## Introduction

Machupo virus (MACV) from the clade B of New World arenaviruses in the *Arenaviridae* family is the etiologic agent of Bolivian hemorrhagic fever (BHF) [1]. Several clade B New World arenaviruses, including MACV, Junin virus (JUNV), Guanarito virus, Sabia virus and Chapare virus, cause hemorrhagic fever diseases in humans in South America [2–4]. The clinical symptoms of BHF are similar to those of Argentine hemorrhagic fever (AHF) caused by JUNV. Both, MACV and JUNV have been declared select agents by U.S. Department of Health and Human Services, and studies with these agents require a biosafety level 4 (BSL4) facility in the USA. The case fatality rate of BHF is 25 to 35% [5,6] and there are no approved treatments or vaccinations. Among human pathogenic arenaviruses, only the live attenuated Candid#1 strain (Cd#1) of JUNV is available as a human vaccine against AHF in Argentine [7,8].

Arenaviruses are bisegmented, negative-stranded RNA viruses [1]. The viral L segment genomic RNA encodes the viral RNA dependent RNA polymerase (L protein) and the small zinc finger protein (Z). The S segment encodes the nucleoprotein (NP) and the glycoprotein precursor (GPC) [1]. The GPC is initially synthesized as a single polypeptide and cleaved into the stable signal peptide (SSP) and GP1/GP2 complex by host signal peptidase. The GP1/GP2 complex is further cleaved into the GP1 and GP2 subunit by host Subtilase SKI-1/S1P [9,10]. The GP1 subunit constitutes a part of GPC ectodomain and binds to the receptor. The pathogenic Clade B New World arenaviruses use human transferrin receptor 1 (hTfR1) as their receptor [11,12], while the Old World arenaviruses and the Clade C New World arenaviruses use alpha-dystroglycan (α-DG) [13,14]. The GP2 subunit contains the remaining part of the ectodomain, a transmembrane domain (TMD) and a cytoplasmic tail (CT) [1,10] (S1 Fig).

**Fig 1:**
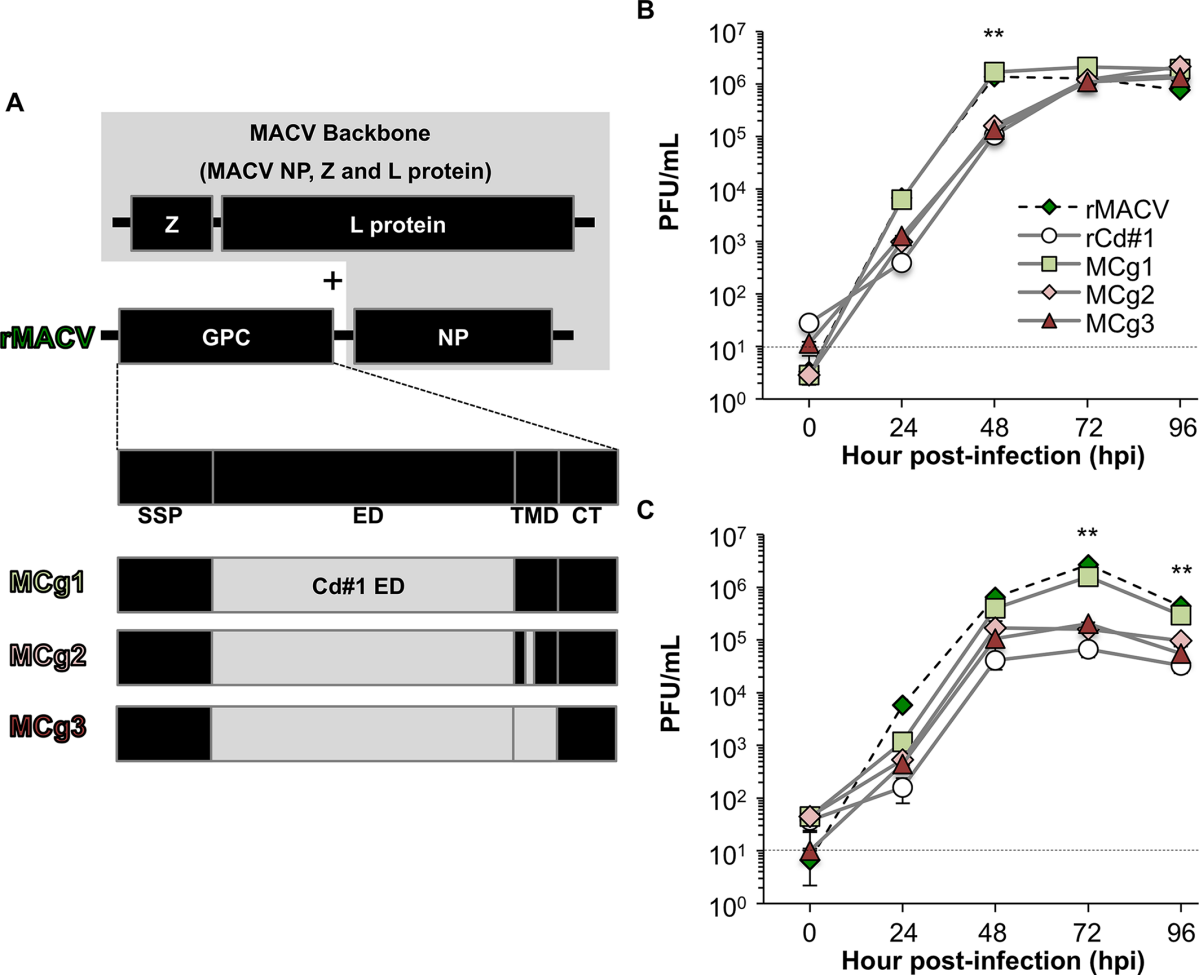
Schematic diagrams and the growth curve of MCg1, MCg2 and MCg3. (A) The ectodomain of GPC alone of rMACV was replaced by the Cd#1 counterpart in rMACV backbone (MCg1), ectodomain of rMACV was replaced by the Cd#1 GPC ectodomain with F427I TMD mutation (MCg2), and the ectodomain and TMD of rMACV GPC were replaced by those of Cd#1 GPC (MCg3). (B) Growth curve of the MCg1, MCg2 and MCg3 were determined in Vero cells (rMACV: N = 3, rCd#1: N = 4, MCg1-3: N = 7). The virus titer of MCg1 was significantly higher than those of rCd#1, MCg2 and MCg3 at 48 hpi in Vero cells (**, *P*<0.01, one-way ANOVA followed by Dunnett's test for MCg1 versus rCd#1, MCg1 versus MCg2 and MCg1 versus MCg3). (C) Growth curve of the MCg1, MCg2 and MCg3 were determined in A549 cells (rMACV: N = 3, rCd#1: N = 4, MCg1-3: N = 6). The virus titer of MCg1 was significantly higher than those of rCd#1, MCg2 and MCg3 at 72 hpi and 96 hpi in A549 cells (**, *P*<0.01, one-way ANOVA followed by Dunnett's test for MCg1 versus rCd#1, MCg1 versus MCg2 and MCg1 versus MCg3). The dashed lines indicate the detection limit. The data of rCd#1 in the Fig 1B and 1C has been published in Fig 1B and 1C respectively, of [16].

We previously found that the GPC of JUNV Cd#1 vaccine strain rendered the pathogenic strain of JUNV and MACV fully attenuated *in vivo* [15,16]. The sequences of TMD of GPC are highly conserved between MACV and JUNV and are crtitical for the virulence of these pathogenic New World arenaviruses. Albarino CG *et al*. and we identified that a single F427I substitution in TMD of JUNV GPC led to attenuation of JUNV in suckling mice following intracranial inoculation [17] and guinea pigs following peripheral inoculation [15], respectively. The mutation alone was apparently not sufficient to fully attenuate JUNV as there is still 10% lethality observed in the suckling mice model [17] and mild diseases manifestations and virus dissemination identified in guinea pigs [15]. For MACV, the equivalent F438I substitution in GP2 TMD also attenuates MACV in IFN-αβ/γ R^-/-^ mice. However, the mutant MACV is genetically unstable and reversion to wild type virus has been identified [18]. All these data collectively indicate that while the TMD is important for virulence, other domains of the GPC may be also important. Sequence comparison of the Cd#1 vaccine strain with the pathogenic JUNV strains indicated several mutations in the ectodomain region of GPC [17] (S1 Fig). Accordingly, we rescued rMACVs with the ectodomain of MACV GPC replaced by its counterpart of Cd#1 either alone (MCg1), in combination with the F427I TMD mutation (MCg2) or with the TMD of Cd#1 (F427I and I431V) (Fig 1A and S1 Fig) and characterized their replication *in vitro* and *in vivo*.

## Methods

### Cells and viruses

African green monkey kidney Vero cells (ATCC, CCL-81) and Baby hamster kidney (BHK-21) cells (ATCC, CCL-10) were maintained in minimal essential medium (MEM) (Life Technologies, Carlsbad, CA) supplemented with 10% FBS (Life Technologies) and 1% Penicillin-Streptomycin (Life Technologies). Human alveolar epithelial cell line A549 cells (ATCC, CCL-185) were maintained in Ham's F-12K Medium (Life Technologies) containing 10% FBS (Life Technologies) and 1% Penicillin-Streptomycin (Life Technologies). The recombinant MACV Carvallo strain (rMACV) [19], recombinant Cd#1 (rCd#1)[20] and MCg1, MCg2 and MCg3 were engineered and then rescued by plasmid transfection previously described. We used the first passage viruses for all infection experiments in this study.

### Animal studies

IFN-αβ/γ R^-/-^ mice (strain 129 mice were backcrossed twice with C57BL/6 mice) were bred and maintained in the ABSL-2 facilities in the Galveston National Laboratory (GNL) at the University of Texas Medical Branch at Galveston. Concurrently performed rMACV and rCd#1 controls were previously described [16]. All animals were implanted with BMDS IPTT-300 transponders (chips) obtained from Bio Medic Data Systems, Inc. (Seaford, DE) at 4–7 days before challenge as previously described [21]. Chips were scanned on the indicated days. To determine if the replacement with ectodomain of Cd#1 GPC leads to attenuation of rMACV, eight to 15 week-old IFN-αβ/γ R^-/-^ mice were challenged by intraperitoneal injection with rMACV, rCd#1, MCg1, MCg2 or MCg3 (10,000 PFU) and monitored for 42 days post-infection (dpi). The animal experiments were performed twice in independent studies. To evaluate the viral dissemination in infected animals, three animals per group were sacrificed at 17 dpi, since MCg1-infected mice started to show symptoms from 17 dpi. Serum, brain, spleen and liver samples were collected for virus titration and IgG ELISA when animals were euthanized or dead. Animals were humanely euthanized at the end of study (42 dpi) or if they became paralyzed or lost more than 20% of the body weight and/or if the body temperature fell below 34°C.

### Construction of S segments for MCg1, MCg2 and MCg3

The S segments of MCg1, MCg2 and MCg3 replaced by the ectodomain of Cd#1 GPC were generated by PCR from three PCR amplicons. The sequences of the primers were listed in Table 1. After digestion with AvrII and gel purification, the DNA of the S segments was inserted into the RNA pol I-driven expression plasmid pRF42 in antigenomic orientation [19,20]. The viral sequencing data for the S segment of MCg1, MCg2 and MCg3 were deposited in DDBJ/EMBL/GenBank (accession number: LC123592, LC123593 and LC123594, respectively).

**Table 1:** Primer set and template.

	S segment fragment region		
	NP side	Cd#1 ectodomain	GPC 5' side
**MCg1 Forward**	cgcacagtggatcctaggcaaag	gcagggaggtcctgctcggaagaagctttcaaaatc	cgcaccggggatcctaggcgattc
**MCg1 Reverse**	cctttgactttagttgacatttgtttctggagcaca	tgtgctccagaaacaaatgtcaactaaagtcaaagg	gattttgaaagcttcttccgagcaggacctccctgc
**MCg1 Template**	rMACV	rCd#1	rMACV
**MCg2 Forward**	cgcacagtggatcctaggcaaag	gcagggaggtcctgctcggaagaagctttcaaaatc	cgcaccggggatcctaggcgattc
**MCg2 Reverse**	cctttgactttagttgacatttgtttctggagcaca	tgtgctccagaaacaaatgtcaactaaagtcaaagg	gattttgaaagcttcttccgagcaggacctccctgc
**MCg2 Template**	F438I	rCd#1	rMACV
**MCg3 Forward**	cgcacagtggatcctaggcaaag	gcagggaggtcctgctcggaagaagctttcaaaatc	cgcaccggggatcctaggcgattc
**MCg3 Reverse**	cacttggtgggtatacccacccatcgacacctcaaa	tttgaggtgtcgatgggtgggtatacccaccaagtg	gattttgaaagcttcttccgagcaggacctccctgc
**MCg3 Template**	rMACV	rCd#1	rMACV

### Ethics statement

All animal studies were performed in facilities accredited by the Association for Assessment and Accreditation of Laboratory Animal Care International (AAALAC International) in accordance to the Animal Welfare Act, NIH guidelines, and US federal law. The Institutional Animal Care and Use Committee at the University of Texas Medical Branch at Galveston approved the study protocol (1208050A).

All the recombinant DNA and viruses were generated upon the approval of the Notification of Use by the Institutional Biosafety Committee at the University of Texas Medical Branch at Galveston. Experiments with rMACV, rCd#1, MCg1, MCg2 and MCg3 were performed in the BSL-4 laboratories at the GNL in accordance with institutional safety guidelines, NIH guidelines and US federal law.

### Growth curve and serial passage

The virus growth curve was determined by measurement of the infectious virus titers in the supernatants of Vero cells and A549 cells infected by viruses at a multiplicity of infection (MOI) of 0.01 for 96 hours post-infection (hpi). To investigate the genetic stability of MCg1, MCg2 and MCg3, the viruses were serially passaged in Vero cells in duplicate as previously described [16,18] The serial passage was performed at an estimated MOI of 0.01 based on Fig 1B every 2 or 3 days. Viral titer was measured by plaque assay on Vero cells as previously described with minor modification [20,22]. The supernatant or serum samples were serially diluted with MEM media containing 2% FBS and incubated on Vero cell monolayers in 12-well plates for 1h at 37°C. Then supernatant was replaced with 0.6% tragacanth overlays (in MEM media containing, 2% FBS and 1% Penicillin-Streptomycin) and incubated for 7 to 8 days at 37°C. Plaques were fixed and stained with 1% crystal violet in 10% formalin.

### Measurement of virus-specific IgG titer by ELISA

Vero cells were infected with rCd#1 or rMACV at MOI = 0.1 and collected at 72 dpi. The infected cells and uninfected cells were lysed with cell lysis buffer (50 mM Tris-HCl, pH 8.0, 300 mM NaCl, 0.5% Triton X-100, 0.5% protease inhibitor cocktail [Sigma-Aldrich, St. Louis, MO]) on ice for 2 h and 30 min. The supernatants were used as the ELISA antigens. ELISA plate was coated with 1:5 diluted cell lysates overnight at 4°C. After washing with PBS buffer containing 0.05% Tween 20 (PBS-T) for three times, the wells were blocked with PBS-T containing 5% bovine serum albumin for 1 h at 37°C and then incubated with 1:100 diluted serum samples for 1 h at 37°C. After washing, horseradish peroxidase-labeled goat anti-mouse IgG antibody (Southern Biotechnology, Birmingham, AL) was added as the secondary antibody. Color reactions were developed using *o*-phenylenediamine dihydrochloride (OPD) (Sigma-Aldrich) for 20 min. The absorbance was measured at 450 nm using a VersaMax ELISA reader (Molecular Devices, Sunnyvale, CA). The optical density (OD) value measured for uninfected cell lysates was subtracted from the OD value of infected cell lysates. Sera from 18 and 16 uninfected mice were used as negative control for rCd#1 and rMACV-specific IgG ELISA, respectively. The cutoff value for positive result was defined as the 5-fold of the SD value of the negative control.

### Plaque reduction neutralization test

Plaque reduction neutralization test (PRNT) was performed to detect neutralizing antibodies against rMACV and rCd#1 as described previously [15]. Heat-inactivated serum or medium samples were serially diluted and incubated with 80 PFU of rMACV or rCd#1 for 1 h at 37°C. The infectious virus titers were obtained on Vero cells by plaque assay as described above. The PRNT_50_ titers were represented as the greatest serum dilution that resulted in 50% reduction in plaque numbers.

### RNA extraction and sequence analysis

RNA was extracted from organs or cells by using Trizol reagent (Life Technologies) and Direct-zol RNA MiniPrep kit (Zymo Research, Irvine, CA) as previously described [16]. The reverse transcription was carried out by using the Superscript III First-Strand Synthesis System (Life Technologies) and random primers according to manufacturer's protocol. cDNAs were amplified by PCR into two and three DNA fragments for viral S and L segments, respectively. The PCR products were purified using a QIAquick PCR purification kit (Qiagen) and directly sequenced using an ABI Prism 3130xl DNA sequencer (Life Technologies). To determine the 5’ and 3’ UTR of the S and L segments, 5’ RACE System for Rapid Amplification of cDNA Ends and the 3’ RACE System for Rapid Amplification of cDNA Ends were used, respectively (Life Technologies).

### Histopathology

Tissues were collected from euthanized or dead animals and fixed in 10% buffered formalin for at least 5 days. Then the tissues were trimmed and embedded in paraffin. Thin sections of the organs (5.0 μm) were stained with hematoxylin and eosin.

### Statistical analysis

Data were analyzed using the Dunnett's post hoc test following a one-way ANOVA, the log rank analysis, and the Mann-Whitney U test. Results were presented as statistically different when the *P* value was <0.05.

## Results

### The replacement of the GPC ectodomain did not affect rMACV growth

The MCg1 containing the ectodomain of Cd#1, the MCg2 containing the ectodomain of Cd#1 and the F427I TMD mutation, and the MCg3 containing the ectodomain and TMD of Cd#1 were engineered and then rescued by plasmid transfection (Fig 1 and S1 Fig). The 3^rd^ residue of GP2 TMD, which related to the virulence, is F438 in MACV, F427 in MCg1 and I427 in MCg2, MCg3 and Cd#1. The growth of MCg1 virus in African green monkey kidney-derived Vero cells, a type I IFN-deficient cell line, were comparable with that of rMACV, while the titers of rCd#1, MCg2 and MCg3 were lower than that of MCg1 at 48 hpi (Fig 1B). Similar results were observed in human alveolar epithelial A549 cells (Fig 1C). The growth kinetics of MCg1 was comparable with that of rMACV and the titer was higher than those of rCd#1, MCg2 and MCg3 at 72 and 96 hpi. These results showed that the replacement of ectodomain of rMACV GPC with that of Cd#1 did not alter the virus growth substantially.

To assess the stability of the TMD sequences in MCg2 and MCg3, these viruses were serially passaged 5 times in Vero cells. No reversions or additional adaptive mutations were detected in any viral genome after 5 cell culture passages.

### Replacement of the ectodomain of rMACV GPC with the counterpart from Cd#1 rendered virus attenuated

**Fig 2:**
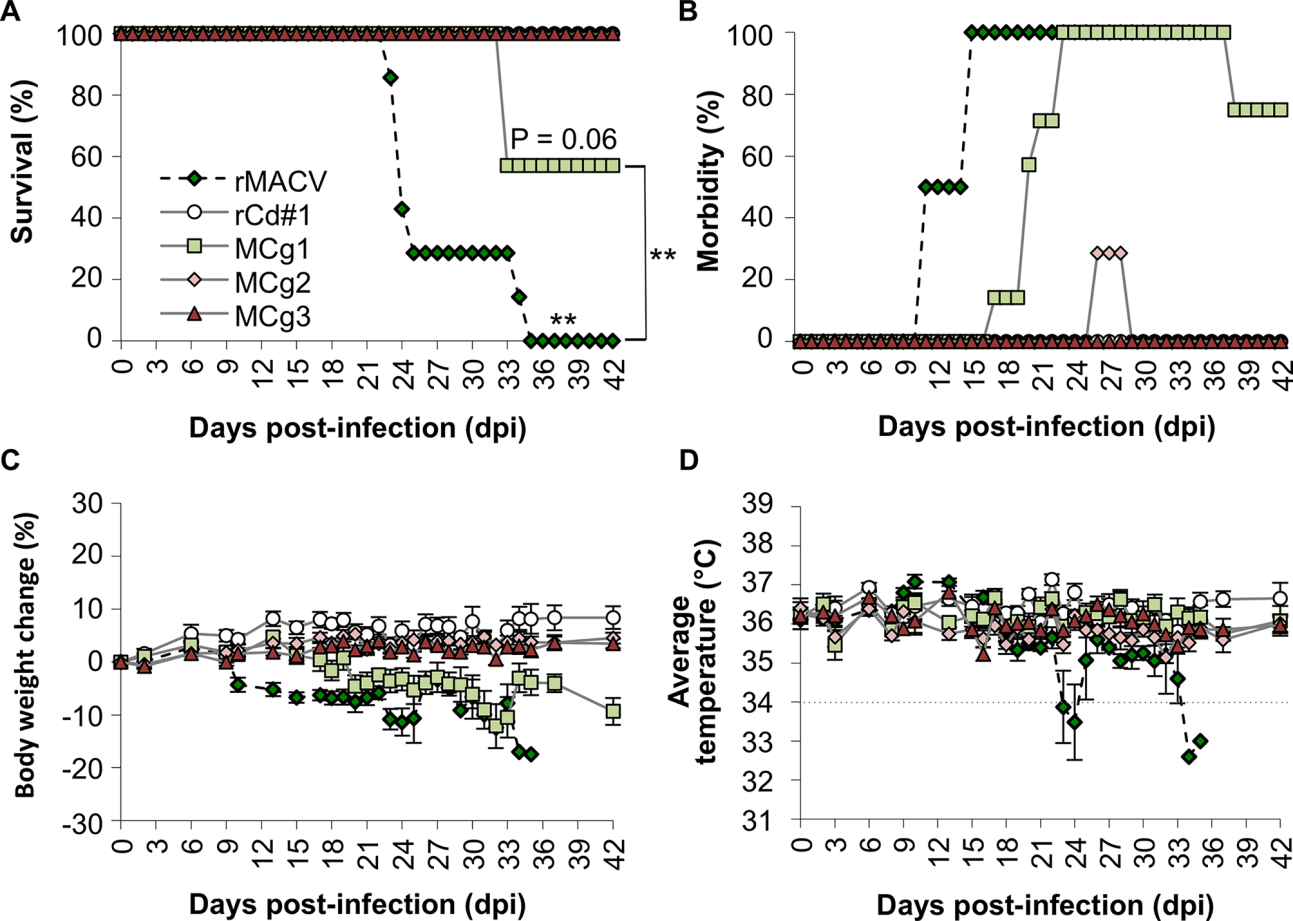
IFN-αβ/γ R^-/-^ mice were infected with 10,000 PFU of rMACV, rCd#1, MCg1, MCg2 and MCg3 by the intraperitoneal route. (A) Survival rate of the IFN-αβ/γ R^-/-^ mice after rMACV, rCd#1, MCg1, MCg2 and MCg3 infections (N = 7 per group except rCd#1, for which N = 6). Statistically significant differences between rMACV- and MCg3-infected groups are indicated by asterisks (**, *P<*0.01 by log rank test). The survival rate of the MCg1-infected group was significantly increased (**, *P<*0.01 for rMACV-infected group versus the MCg1-infected group). No significant difference was observed between MCg1- and MCg3-infectred groups (*P* = 0.06). (B) Clinical symptoms were monitored daily. All rMACV-infected animals showed disease such as scruffy coats and hunched postures at 11 to 15 dpi, while MCg1-infected animals showed delayed onset of the symptoms by 2–12 days. Two of mice infected MCg2 showed mild scruffy coats at 26 dpi to 28 dpi. (C) Body weight changes were monitored on the indicated days. Error bars indicate the SEM (N = 7 per group except rCd#1, for which N = 6). Loss of 5% of body weight in MCg1-infected mice was delayed by 6 to 13 days than in rMACV group. (D) Body temperatures were monitored on the indicated days. While six of seven rMACV-infected animals showed hypothermia (below 34°C) at 1 to 3 days prior to death, no exhibited hypothermia in groups of rCd#1-, MCg1-, MCg2- and MCg3-infected animals. The dots line indicates 34°C. The rMACV and rCd#1 were inoculated into IFN-αβ/γ R^-/-^ mice as a positive control and negative control, respectively, at the same time with MCg1, MCg2 and MCg3. The data of rMACV and rCd#1 in the Fig 2A, 2C and 2D has been published in Fig 2A, 2B and 2C respectively, of [16].

To evaluate their pathogenicity in our established lethal MACV murine model, MCg1, MCg2 and MCg3 were intraperitoneally inoculated into IFN-αβ/γ R^-/-^ mice. As expected, all animals infected with rMACV succumbed to infection or reached a humane study endpoint by 35 dpi, which was also reported in the previous study [16]. However, only 3 out of seven mice succumbed to MCg1 infection at 33 dpi (Fig 2A). The survival rate of the MCg1-infected group was significantly increased when compared with rMACV-infected group (*P<*0.01, log rank test). All rMACV-infected animals developed disease manifestations such as scruffy coats and hunched postures, at 11 to 15 dpi as described in the previously published work [16]. On the other hand, the onset of the symptoms in MCg1-infected animals was delayed by an average of 7.6 days (range, 2–12 days)(Fig 2B). Similar tendency was observed in body weight changes. More than 5% weight loss was first observed at an average of 13.7 dpi (range, 10–18 dpi) in rMACV-infected animals and at an average of 22.2 dpi (range, 15–31 dpi) in MCg1-infected animals (Fig 2C). Hypothermia and imbalance were shown only in rMACV-infected mice at 1 to 3 days before death (Fig 2D). On the other hand, none of the rCd#1-, MCg2- and MCg3-infected animals succumbed to infection. Only two of 7 animals infected with MCg2 showed mild symptoms, such as mild scruffy coats at 26 dpi for 3 days. However, no body weight loss and other symptoms were observed in MCg2-infected as well as in rCd#1 and MCg3-infected animals. These data indicate that the ectodomain of Cd#1 GPC in combination with the TMD mutation led to more potent attenuation than the Cd#1 ectodomain alone.

**Fig 3:**
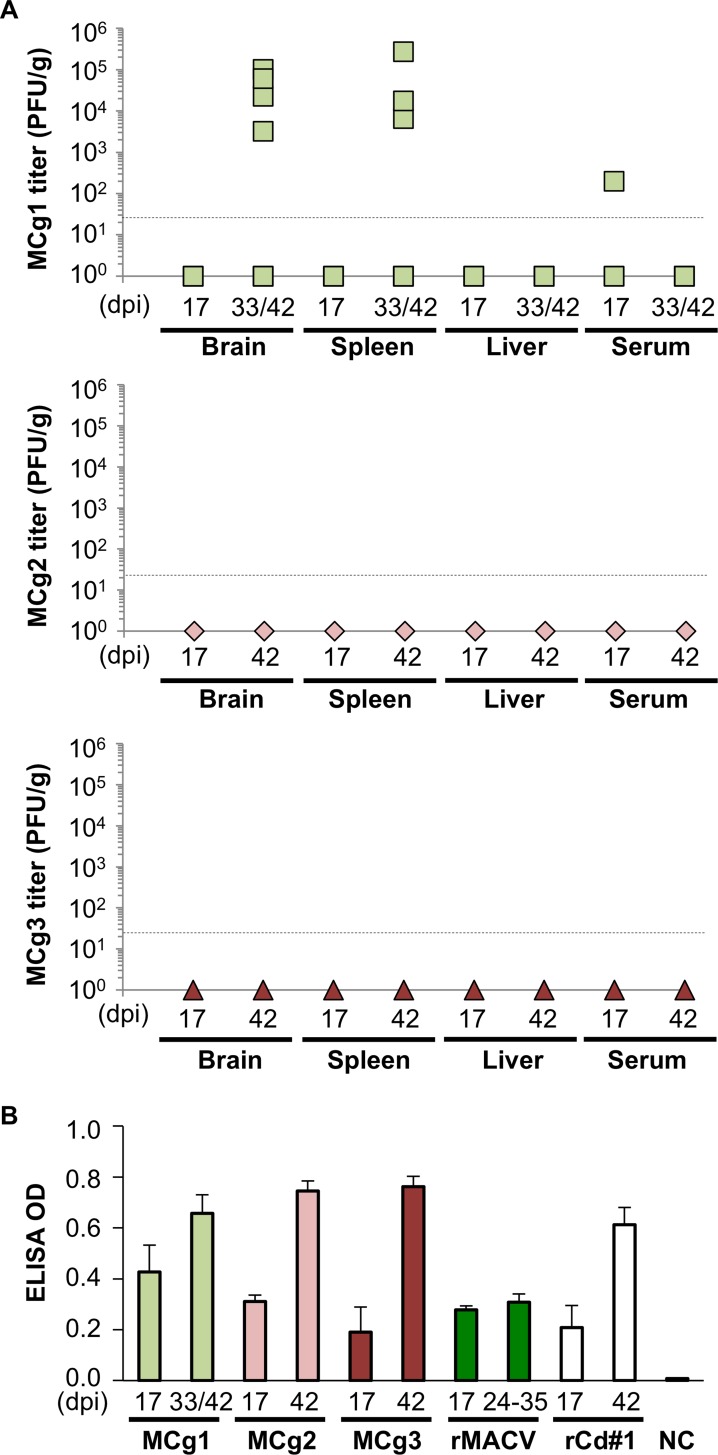
Viral titers in organs and rCd#1-specific IgG induction after infection. (A) Viral titer was determined in brain, spleen and serum for MCg1, MCg2 and MCg3. Dashed line indicates the minimum detection limit. (B) ELISA measurement of IgG was performed to detect immune response in mice after infection. The OD value obtained from uninfected cell lysates was used as the negative control for cell lysates and was subtracted from the OD value of infected cell lysates. All tested samples, except for two samples collected at 17 dpi (from the MCg3-infected group and rCd#1-infected group), were positive. Error bars indicate the SEM (N = 3 for 17 dpi and N = 5 for 33/42 dpi or 42 dpi). The data of rMACV and rCd#1 in the Fig 3B has been published in Fig 2E of [16].

In the IFN-αβ/γ R^-/-^ mouse model, rMACV disseminates systemically and penetrates the CNS of infected mice [16,19]. Since MCg1 was attenuated to some extent based on diseases manifestations in the murine model, we investigated the dissemination of MCg1 to the brain, liver, spleen and its presence in serum (Fig 3A). Whereas rMACV was detected in brain, spleen, liver and serum samples of all the mice at the terminal stage (24 dpi to 35 dpi) as shown in our previously published data [16], MCg1 was detected in some of the brains (4 of 7 mice at 33 and 42 dpi), spleens (3 of 7 mice at 33 and 42 dpi) and serum samples (1 of 3 mice at 17 dpi) (Fig 3A). Compared with the previously published data of rMACV [16], the average MCg1 titer (in 4 positive cases) in brains was 340-fold lower at 33 dpi or 42 dpi than that of rMACV-infected animals at the terminal stage (24 dpi to 35 dpi). The average titer in spleens was comparable between rMACV- and MCg1-infected animals. The titers in livers of MCg1-infected animals were below the detection limit. These results suggested a restricted ability of MCg1 to disseminate as compared to rMACV. No infectious virus was detected in the organs and sera from rCd#1-, MCg2- and MCg3-infected animals (Fig 3A) [16]. Altogether, these findings support the hypothesis that the replacement of the ectodomain of MACV GPC led to greatly impaired dissemination of the viruses to the brains and livers.

To assess the host humoral immune response in the infected animals, an indirect IgG ELISA was carried out using the lysates prepared from virus-infected cells. All samples tested were IgG positive for both rCd#1 and rMACV, except for one from MCg3-infected animal at 17 dpi and one from rCd#1-infected animal at 17 dpi (Fig 3B and S2 Fig). The antiviral IgG levels were comparable among serum samples from MCg1, MCg2 and MCg3-infected animals, indicating that MCg2 and MCg3 elicited similar levels of immune responses as MCg1, although viruses were not detectable in the organs of MCg2 and MCg3 infected animals. Since almost all animals produced antibodies against rCd#1 and rMACV based on ELISA, neutralizing antibody titers against rCd#1 and rMACV were determined by PRNT_50_ assay (Table 2). MCg1 infection in general induced high levels of neutralizing antibody against rCd#1 and relatively low levels of neutralizing antibody against rMACV, suggesting that the antibodies against the ectodomain of Cd#1 GPC could not efficiently neutralize MACV. In rMACV-infected animals, the neutralizing antibody levels against rCd#1 and rMACV were both lower than those in MCg1-infected mice (Table 2) [16]. In MCg2 and MCg3-infected animals, high titers of neutralizing antibody against rCd#1 were found, but the neutralizing antibodies against rMACV were all below the detection level (Table 2). These data suggest the inability of MCg2 and MCg3 to induce detectable neutralizing antibody response against rMACV.

**Table 2:** PRNT_50_ titers against rCd#1 and rMACV (serum dilution 1:30–1:960).

MCg1-infected	PRNT_50_ titer to		MCg2-infected	PRNT_50_ titer to		MCg3-infected	PRNT_50_ titer to	
mice	rCd#1	rMACV	Mice	rCd#1	rMACV	mice	rCd#1	rMACV
# 1	1:480 (d33^1^)	1:30 (d33)	# 8	>1:960	ND	# 15	1:480	ND
# 2	>1:960	1:240	# 9	1:480	ND	# 16	>1:960	ND
# 3	>1:960 (d33)	ND^3^ (d33)	# 10	>1:960	ND	# 17	>1:960	ND
# 4	>1:960	1:120	# 11	>1:960	ND	# 18	>1:960	ND
# 5	>1:960 (d33)	1:60 (d33)	# 12	>1:960	ND	# 19	>1:960	ND
# 6	>1:960	1:120	# 13	>1:960	ND	# 20	1:240	ND
# 7	>1:960	ND	# 14	>1:960	ND	# 21	>1:960	ND
Geometric mean^2^	>1:869.5	1:90.9	Geometric mean	>1:869.5	ND	Geometric mean	>1:713.3	ND

Table 2: The PRNT titers to rCd#1 and rMACV of the serum samples from MCg1-, MCg2- and MCg3-infected mice were measured.

1) Numbers in parentheses indicates the day after infection. If not indicated, the subject survived until 42 dpi.

2) Geometric mean of the PRNT titers. The geometric mean of the PRNT titers of sera from rMACV-infected animals is 1:60.0 against rCd#1 and is below the detection level against rMACV. The geometric mean of the PRNT from rCd#1-infected animals is >1:762.0 against rCd#1 and not detectable level against rMACV. These PRNT titers against rMACV from rMACV- and rCd#1-infected control groups were reported previously [16].

3) ND: not detectable, <1:30

**Fig 4:**
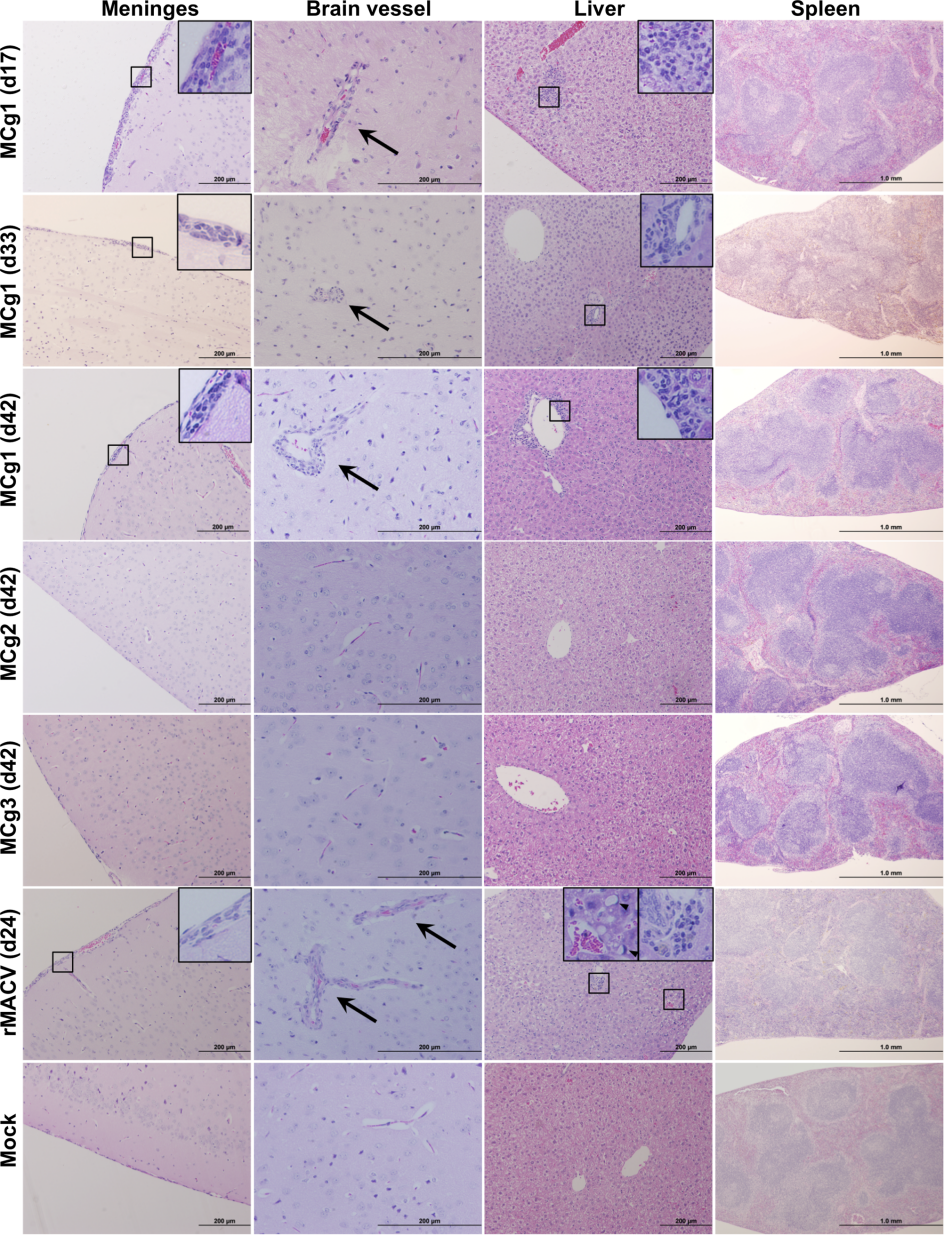
Histopathological changes in the brain, spleen, and liver from infected mice at 17 dpi, the terminal stage and 42 dpi. Meningitis and perivascular cuffing (Arrow) were observed in the brains of MCg1-infected mice at 17 dpi, the terminal stage (33 dpi), the end of the study (42 dpi) and the brain of rMACV-infected mice at the terminal stage (24 dpi). Periportal infiltrates or focal inflammation were present in the livers of MCg1-infected animals at 17 dpi, 33 dpi and 42 dpi. Periportal infiltrates and microvesicular steatosis (Arrowhead) were observed in the liver of rMACV-infected animal at 24 dpi. Reactive white pulp hyperplasia was mildly disturbed in the spleen of MCg1-infected animal at 33 dpi and moderately in the spleen of rMACV-infected animal at 24 dpi. No significant histological change was observed in MCg2- and MCg3-infeceted animals at 42 dpi. Magnifications, x4 (Spleen), x10 (Meninges and Liver), x20 (Brain Vessel) and x40 (insets).

Histopathological examination was performed by staining the paraffin embedded sections with hematoxylin and eosin (H&E). In brains, meningitis and perivascular cuffing were frequently detected in MCg1-infected mice and rMACV-infected mice but not in rCd#1-, MCg2- and MCg3-infected mice (Fig 4, S3 Fig and Table 3). Focal inflammation and periportal infiltrates in liver were observed in some of MCg1-infected animals and most of rMACV-infected animals. One of the MCg2-infected mice developed mild symptoms (Fig 2B) and had a small focal inflammation in 2 sections of the liver. Microvesicular steatosis of liver was frequently observed only in rMACV-infected animals. Reactive white pulp hyperplasia was characterized in spleens of some of MCg1-infected animals and most of rMACV-infected animals. Taken together, infection with MCg1 expressing the ectodomain of Cd#1 GPC caused milder histopathological changes when compared with rMACV, meanwhile the MCg2 and MCg3 containing additional changes in TMD were highly attenuated and no organ lesions were observed.

**Table 3:** Frequency of histopathological observation.

	Brain		Liver			Spleen
Group and days	Meningitis	Perivascular cuffing	Focal inflammation	Perivascular mononuclear infiltrates	Microvesicular steatosis	Reactive white pulp hyperplasia
MCg1 d17 (N = 3)	3	3	2	0	0	0
MCg1 Terminal (N = 3)	3	2	1	0	0	2
MCg1 d42 (N = 4)	4	4	1	1	0	2
MCg2 d17 (N = 3)	0	0	0	0	0	0
MCg2 d42 (N = 7)	0	0	1	0	0	0
MCg3 d17 (N = 3)	0	0	0	0	0	0
MCg3 d42 (N = 7)	0	0	0	0	0	0
rMACV d17 (N = 3)	3	3	3	3	1	3
rMACV Terminal (N = 7)	5	7	4	6	7	6
rCd#1 d17 (N = 3)	0	0	0	0	0	0
rCd#1 d42 (N = 6)	0	0	0	0	0	0

## Discussion

To investigate the potential role of the ectodomain of Cd#1 GPC in the attenuation of rMACV/Cd#1-GPC, rMACV expressing Cd#1 ectodomain alone (MCg1), along with the F427I TMD mutation (MCg2) or with the TMD of Cd#1 (MCg3) were developed using reverse genetics system. Here we showed for the first time that the replacement of MACV GPC ectodomain with that of Cd#1 renders MACV partially attenuated as compared with rMACV/Cd#1-GPC, suggesting that not only the ectodomain but other domains in Cd#1 GPC might also contribute to the higher level of attenuation rMACV/Cd#1-GPC in the animal model. Additionally, rMACV/Cd#1-GPC, which contains the entire Cd#1 GPC in MACV backbone, was able to induce higher levels of anti-rMACV-neutralizing antibody (geometric mean PRNT titer, 1:161) [16] than MCg1 (geometric mean PRNT titer, 1:90.9) in infected animals. This result also suggested that the SSP, TMD and CT domains of Cd#1 GPC could enhance the induction of antibodies that neutralize rMACV.

MCg1-infected animals showed a significant reduction in mortality and prolonged survival time when compared to the rMACV group (Fig 2A). The onset of the disease was delayed and milder in MCg1-infected mice than in rMACV-infected animals (Fig 2B and 2C). The virus titer of MCg1 in brains and livers was also lower than those of rMACV [16] (Fig 3A). Consistent with these findings, the pathological changes in livers and spleens of MCg1-infected animals were less severe than those of rMACV-infected animals (Fig 4 and Table 3). These findings indicated that the ectodomain of Cd#1 GPC alone could render MACV partially attenuated, however, other domain such as TMD in Cd#1 GPC is also required to achieve full attenuation of the virus.

MCg1 infection was able to elicit humoral immune response, as evidenced by the production of virus specific IgG and the neutralizing antibodies against rMACV and rCd#1 (Fig 3B, S2 Fig and Table 2). MCg1 infection of animals caused a potent anti-rMACV-neutralizing antibody response (geometric mean PRNT titer, 1:90.9), while rMACV infection did not induce measurable anti-rMACV-neutralizing antibody response. Interestingly, both viruses induced similar levels of rMACV-specific IgGs as measured by indirect ELISA (S2 Fig). These data suggest that MCg1 could induce protective immune response, which might partly explain the attenuation of MCg1. Further studies on the mechanism of attenuation of MCg1 are needed.

In MCg2-infected mice, only two animals transiently developed mild scruffy coats for 3 days (Fig 2B), and one of the mice had a small focal inflammation in the liver (Table 3). No clinical symptoms were observed in other MCg2- and MCg3-infected animals. Furthermore, MCg2 and MCg3 viruses were not detected in infected animals both at 17 dpi and 42 dpi (Fig 3B). Humoral immunity plays a pivotal role in protection from New World arenavirus infection. For example, immune plasma treatment with high levels of neutralizing antibody effectively reduced the fatality rate of AHF and BHF in humans and in nonhuman primates (NHPs), respectively [23,24]. Although the MCg2 and MCg3 viruses are avirulent in the murine model, the PRNT_50_ data indicated that both viruses elicited very low level of neutralization antibodies against rMACV (Table 2). Therefore, unlike the rMACV/Cd#1-GPC [16], the MCg2 and MCg3 may not efficiently protect the host from MACV challenge. However, additional testing in fully immunocompetent hosts, such as outbread guinea pigs, may be needed to better understand their potential to induce protection.

Our results suggested the importance of all GPC domains in virus propagation. The growth kinetic of MCg1 that has the SSP, TMD and CT domains of MACV, was comparable to that of rMACV both in Vero cells and A549 cells (Fig 1B and 1C). The titers of MCg2 and MCg3 that have the F427I mutation in TMD, were lower than that of MACV in Vero cells at 24 to 48 hpi and more notably in A549 cells at 24 to 96 hpi, indicating that the F427I mutation in TMD subtly affected the virus growth *in vitro*. Our data is consistent with others’ results showing the role of GPC TMD in arenavirus infection *in vitro*. A previous report demonstrated that the F427I mutation leads to reduced infectivity of Cd#1 in a mini-genome system and proposed that the substitution causes a structural defect that destabilizes the metastable conformation at neutral pH and decreases viral infectivity [25]. Another study has indicated that the interaction between SSP and TMD of JUNV GPC might be important in virus fusion with host membrane [26]. In addition to the TMD, the SSP and CT domains might also contribute to the relatively lower replication of MCg2 and MCg3 in cultured cells. A study on chimeric Cd#1 JUNV expressing LASV GPC has demonstrated that whilst the ectodomains of Cd#1 and LASV GPCs are exchangeable, the virus with heterologous SSP, TMD and CT could not be rescued, indicating that homologous SSP, TMD and CT is essential for production of infectious chimeric JUNV/LASV virus [27]. Accordingly, it is possible that the less efficient multiplications of MCg2 and MCg3 might be also due to incompatibility of heterologous SSP, TMD and CT domains. The homologous SSP, TMD and CT of MACV in MCg1 and rMACV might facilitate the optimal multiplication of viruses, meanwhile the heterologous SSP (MACV), CT (MACV) and TMD (Cd#1) in MCg2 and MCg3 might affect virus multiplication in cultured cells. While the impact of the SSP, TMD and CT domains on virus fitness *in vivo* are not investigated directly, it is possible that TMD mutations or the incompatibility of heterologous domains might more profoundly affect virus infection *in vivo* and augment the attenuation of MCg2 and MCg3 in our mouse model. This might help explain our data that neither virus nor neutralizing antibody to rMACV could be detected in MCg2 and MCg3-infected animals, which is in contrast to the MCg1 group. Furthermore, other studies suggest that SSP interacts with the ectodomain of GP2 [26]. Therefore it is also possible that the mismatch of MACV SSP with the Cd#1 GP2 in MCg1, MCg2 and MCg3 might contribute to the attenuation. One other possibility for lower titers of MCg2 and MCg3 is potentially the less efficient budding and virion formation or by higher ratio of viral genome equivalents to PFU.

Taken together, we demonstrated in our study that the ectodomain of Cd#1 GPC contributed to the attenuation in rMACV/Cd#1-GPC, although the ectodomain alone is not sufficient for complete attenuation. Our new findings could shed insight into the development of vaccine candidates not only for MACV but also for other New World arenaviruses.

## Supporting Information

S1 Fig Amino acid sequence alignment of MACV, JUNV Romero strain and Cd#1 GPC. Each domain region is shown over the sequence. Amino acid sequences of MACV, JUNV Romero strain and Cd#1 GPC (DDBJ/EMBL/GenBank accession number: AIG51558.1, AAT40447.1 and AAU34180.1, respectively) were aligned using Genetyx-Mac Ver.13 (Genetyx Corporation, Tokyo, Japan). The single amino acid on TMD involving the pathogenicity is displayed by upward arrow (aa 438 for MACV and aa 427 for JUNV).
(TIF)

S2 Fig rMACV-specific IgG induction after infection. ELISA measurement of IgG was performed to detect immune response in mice after infection. The OD value obtained from uninfected cell lysates was used as the negative control for cell lysates and was subtracted from the OD value of infected cell lysates. All tested samples, except for two samples collected at 17 dpi (from the MCg3-infected group and rCd#1-infected group), were positive. Error bars indicate the SEM (N = 3 for 17 dpi and N = 5 for 33/42 dpi or 42 dpi).
(TIF)

S3 Fig Histopathological changes in the brain, spleen, and liver from infected mice at 17 dpi. No significant histological change was observed in MCg2- and MCg3-infeceted animals at 17 dpi and in rCd#1-infeceted animals at 17 dpi and 42 dpi. Magnifications, x4 (Spleen), x10 (Meninges and Liver) and x20 (Brain Vessel).
(TIF)

S1 File The viral sequencing data in FASTA format for the S segment of MCg1, MCg2 and MCg3. The viral sequencing data for the S segment of MCg1, MCg2 and MCg3 were deposited in DDBJ/EMBL/GenBank (accession number: LC123592, LC123593 and LC123594, respectively).
(PDF)
