# Supplementary figures and images for: The Ectodomain of Glycoprotein from the Candid#1 Vaccine Strain of Junin Virus Rendered Machupo Virus Partially Attenuated in Mice Lacking IFN-αβ/γ Receptor
Source: PLoS Negl Trop Dis. 2016 Aug 31;10(8):e0004969. doi: 10.1371/journal.pntd.0004969 (PMC5006991; doi:10.1371/journal.pntd.0004969)

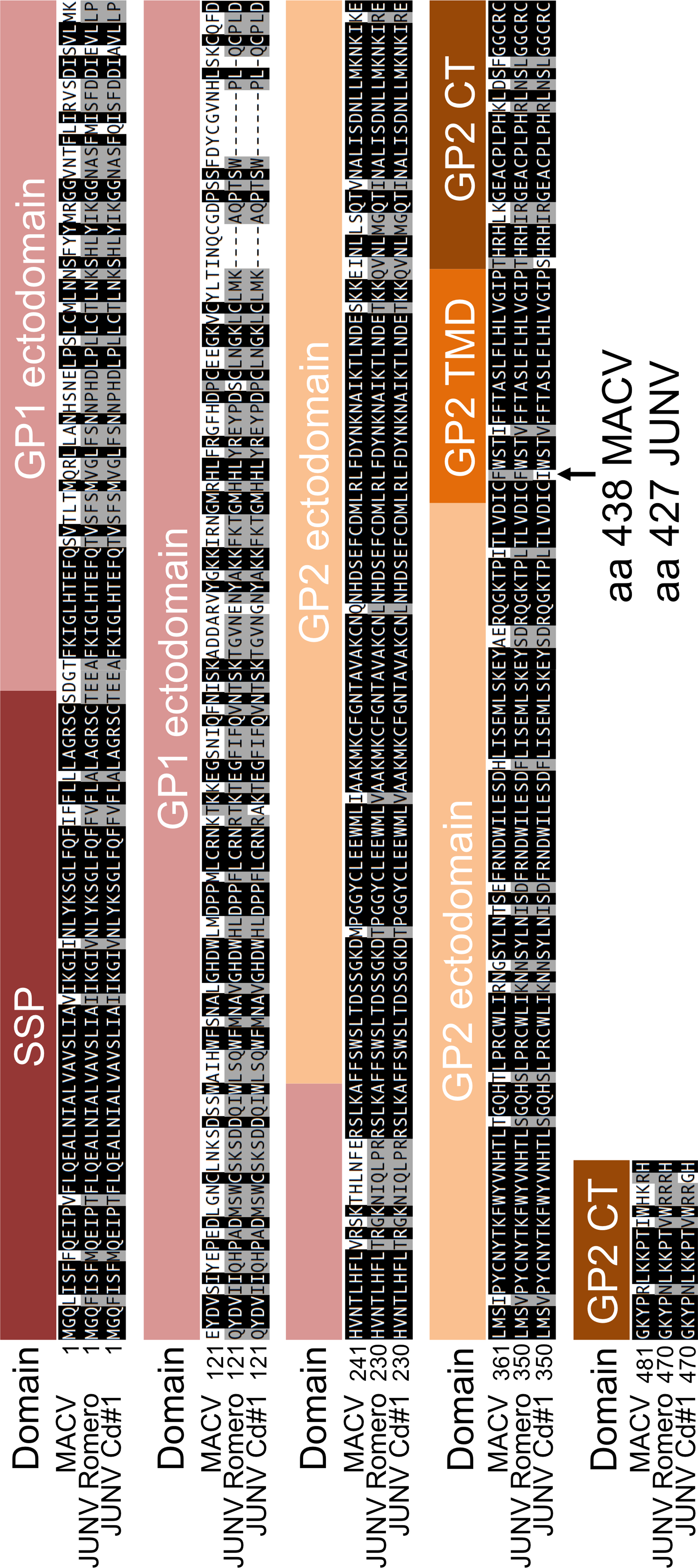

Supplement: S1 Fig — Each domain region is shown over the sequence. Amino acid sequences of MACV, JUNV Romero strain and Cd#1 GPC (DDBJ/EMBL/GenBank accession number: AIG51558.1, AAT40447.1 and AAU34180.1, respectively) were aligned using Genetyx-Mac Ver.13 (Genetyx Corporation, Tokyo, Japan). The single amino acid on TMD involving the pathogenicity is displayed by upward arrow (aa 438 for MACV and aa 427 for JUNV). (TIF) [file pntd.0004969.s001.tif]

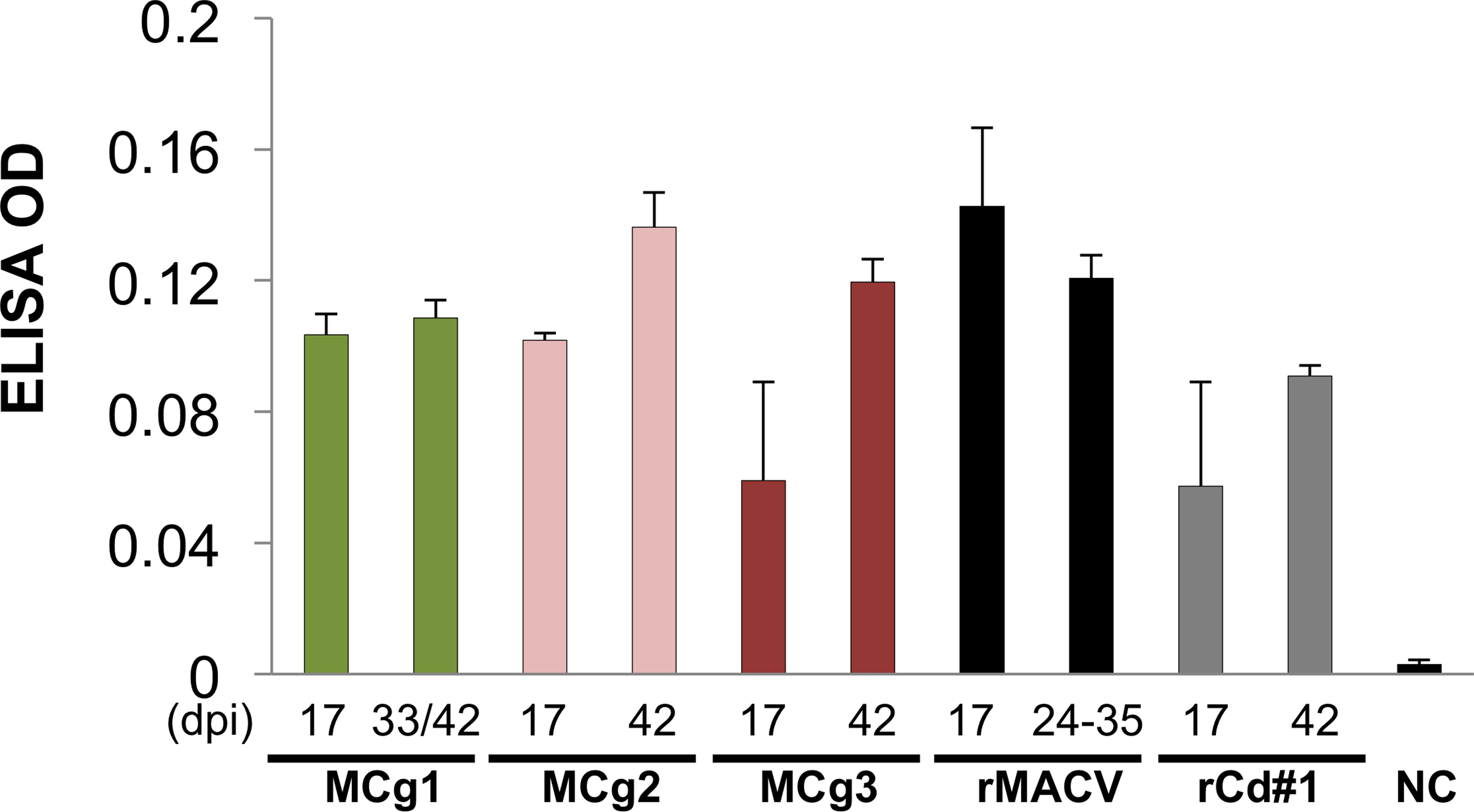

Supplement: S2 Fig — ELISA measurement of IgG was performed to detect immune response in mice after infection. The OD value obtained from uninfected cell lysates was used as the negative control for cell lysates and was subtracted from the OD value of infected cell lysates. All tested samples, except for two samples collected at 17 dpi (from the MCg3-infected group and rCd#1-infected group), were positive. Error bars indicate the SEM (N = 3 for 17 dpi and N = 5 for 33/42 dpi or 42 dpi). (TIF) [file pntd.0004969.s002.tif]

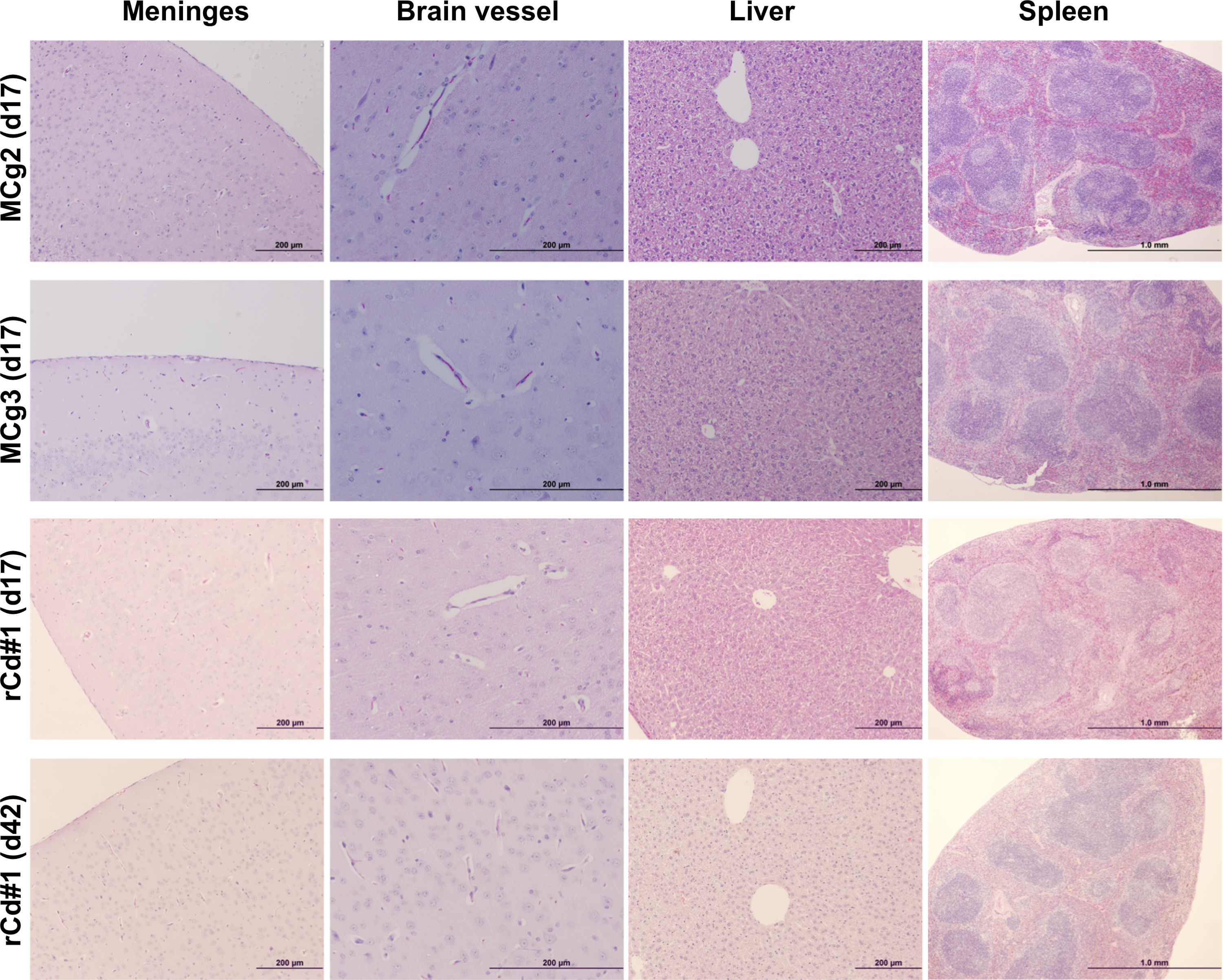

Supplement: S3 Fig — No significant histological change was observed in MCg2- and MCg3-infeceted animals at 17 dpi and in rCd#1-infeceted animals at 17 dpi and 42 dpi. Magnifications, x4 (Spleen), x10 (Meninges and Liver) and x20 (Brain Vessel). (TIF) [file pntd.0004969.s003.tif]
