# Supplementary material for: The Ectodomain of Glycoprotein from the Candid#1 Vaccine Strain of Junin Virus Rendered Machupo Virus Partially Attenuated in Mice Lacking IFN-αβ/γ Receptor
Source: PLoS Negl Trop Dis. 2016 Aug 31;10(8):e0004969. doi: 10.1371/journal.pntd.0004969 (PMC5006991; doi:10.1371/journal.pntd.0004969)
Supplement: S1 File — The viral sequencing data for the S segment of MCg1, MCg2 and MCg3 were deposited in DDBJ/EMBL/GenBank (accession number: LC123592, LC123593 and LC123594, respectively). (PDF) [file pntd.0004969.s004.pdf]

DDBJ/EMBL/GenBank (accession number: LC123592)
>MCg1_S/LC123592.seq
CGCACCGGGGATCCTAGGCGATTCTTGATCGCGCTTATTAGCTAACCAATTTAATTTGGT
GTTGAAGTGTTGACACGCTCTCTAACACATGGGGCAGCTTATCAGCTTCTTTCAGGAGAT
TCCTGTTTTTCTACAGGAAGCTCTGAACATCGCTTTAGTGGCTGTTAGTCTCATAGCTGT
CATCAAAGGCATCATTAACCTTTACAAAAGTGGTCTCTTCCAGTTCATCTTCTTTCTCCT
CCTAGCAGGGAGGTCCTGCTCGGAAGAAGCTTTCAAAATCGGACTGCACACTGAGTTCCA
GACTGTGTCCTTCTCAATGGTGGGTCTCTTTTCCAACAATCCACATGACCTACCTTTGTT
GTGTACCTTAAACAAGAGCCATCTTTACATTAAGGGGGGCAATGCTTCATTTCAGATCAG
CTTTGATGATATTGCAGTATTGTTGCCACAGTATGATGTTATAATACAACATCCAGCAGA
TATGAGCTGGTGTTCCAAAAGTGATGATCAAATTTGGTTGTCTCAGTGGTTCATGAATGC
TGTGGGACATGATTGGCATCTAGACCCACCATTTCTGTGTAGGAACCGTGCAAAGACAGA
AGGCTTCATCTTTCAAGTCAACACCTCCAAGACTGGTGTCAATGGAAATTATGCTAAGAA
GTTTAAGACTGGCATGCATCATTTATATAGAGAATATCCTGACCCTTGCTTGAATGGCAA
ACTGTGCTTAATGAAGGCACAACCTACCAGTTGGCCTCTCCAATGTCCACTCGACCACGT
TAACACATTACACTTCCTTACAAGAGGTAAAAACATTCAACTTCCAAGGAGGTCCTTGAA
AGCATTCTTCTCCTGGTCTTTGACAGACTCATCCGGCAAGGATACCCCTGGAGGCTATTG
TCTAGAAGAGTGGATGCTCGTAGCAGCCAAAATGAAGTGTTTTGGCAATACTGCTGTAGC
AAAATGCAATTTGAATCATGACTCTGAATTCTGTGACATGTTGAGGCTCTTTGATTACAA
CAAAAATGCTATCAAAACCCTAAATGATGAAACTAAGAAACAAGTAAATCTGATGGGGCA
GACAATCAATGCCCTGATATCTGACAATTTATTGATGAAAAACAAAATTAGGGAACTGAT
GAGTGTCCCTTACTGCAATTACACAAAATTTTGGTATGTCAACCACACACTTTCAGGACA
ACACTCATTACCAAGGTGCTGGTTAATAAAAAACAACAGCTATTTGAACATCTCTGACTT
CCGTAATGACTGGATATTAGAAAGTGACTTCTTAATTTCTGAAATGCTAAGCAAAGAGTA
TTCGGACAGGCAGGGTAAAACTCCTTTGACTTTAGTTGACATTTGTTTCTGGAGCACAAT
TTTCTTCACAGCATCATTGTTCCTTCATCTAGTTGGA-ATACCCACCCATCGACACCTCA
AAGGCGAAGCCTGTCCTTTGCCTCATAAGCTGGACAGCTTCGGAGGTTGTAGATGTGGCA
AATATCCCAGATTGAAGAAACCCACCATCTGGCACAAAAGACATTAAACACAGCCAAGAC
CCCTGCCGACCCGGGCCCAGCCCGGGTCGGCGGGGCCCCCCAGTCCGCGGCTCTGCCGCG
GACTGGGGAGGCACTGCTTACAGTGCAAAGGCTGCCTTGGGTAGAAATAGAAGGCTGAGA
GGTAAAAGTGCCACTGGTTCTTCATCTACCATTTTGCCATCCATCACTGATTGATACATG
ATACAATCCAACAGAGCACAGTGAGGTTCACCAGAACTCAATTTGAATGGAGAGTCTTTT
TCTCTCTTTTTCTTGTTGACAACAACTCCATTATGATGTTTGCAAAGATGTCCAAACTTC
TCCCAAATTTGCTGATCATAGAGTCTTGCTTGATCAGATGTGAGTTTCACATCTACCAGC
TTTAAATCCTTCCGTCCATGAATGTCTAAAAGTTTCCTTATGTCGTCAGAACCTTGAGTT
GTAATCACCATATCTTGTGGTAGTAAACCTATTACATAACTTAATACTCCTGGCATTGCA
TCCTCGATGTCTTGCATCAAGATGCCATGTGAATGTCTGCTGCCATTTTTGAAGCCCTTC
TCATCATGTGGCTTTCTAAAACAATGAATATAATGCTTGTTGGCTGGCTGATATAGTGCC
AATTCCACAGGGTCTGTTGGAGGCCCTTCAATGTCAAGCCACAGTGTATTAGTGGGGTCT
AACTTCTTTACAGCCTCTTTAATCACTGCTTCTTGCATTTCAGTCAGGTTTGCTAGTCTT
AGATTCTGACCGTTTTTCTCGGGTTGTCTCGGTCCAACTTGAGGTTTCTTTGTTAGATCA
ACACTTGTGTTGTCCCAAGATCTCCCAAGAATTTGTGATCTGGAACCAATGTAAGGCCAC
CCGTCACCTGAGAGACACAATTTATACAGAAGGTTCTCATAAGGATTTCGATTCCCAGGT
TTCTCGTCTATAAACATCCCCTCTTTTCGCTTGACTTGCAGAGTTGATTTAATCAAGCTG
GAGAAATTATCAGGAGTGACCCTAATTGTTTCCAGCATGTTCCCACCGTCAAGTATTGAG
GCGCCAGCTTTCACAGCAGCTGACAGACTGAAGTTATAGCCAGAGATGTTGATGGAGCTC
TCGTCCTTAGTTACGATCTGCAAGCATTCGTGCTCTAGAGTTAGCTTGTCAAGGTCATTT
AAATTCGGATATTTAACAGTGTATAACAGGCCCAGAGATGTCAACGCTTGGACCACACTA
TTCATCGTTTCTCCACCCTGGACAGTCATACAAGCGATTGTGAGAGCTGGCATGGAACCA
AACTGATTATTCAGTTTTGATGGATCTGAGACATCCCAGATTCTAACCACACCATTTCCA
GCACCTCTCTGCTGTTGGAATCCCAGGGTCTTCAAGATTTCTGACCTCTTTGTCAGTTGC
AGCTGTGACAAATTACCCATGTACAGCCCTTGGGGACCTGACTCAGTCCTCTTTATCTTG
TTCTTCAGTTTTTCCAAGTCTGATGCAAGCTCCATCAATTCATCTCTCCCCAGATCTCCA
ATCTTGAATATGGTGTTTTTCTGGATGCTTTTCATAGACATGAGCCTATCCACTTCTTTG
TTTAAATCCCTCAGCTTGTTCAGATCCTCTTCACCCCTTTTGTTCTTTCTGAGAGCCCTC
TGCACTTGTGAAACCTGGTTGAAGTCGATGCTGTCAGCTATGAGCTTGGCATCTTTCAAC
ACATCTGTTTTCACAGTGTGGGTGAACTGACTCAGGCCTCTTCTCAGTGACTGAGTCCAC
CGAAAGCTGGGAATTTCCTTGGAGTGAGCCATAGTGGCTAATAACCAAAGGATTTGAAAT

AAAGCTCAGTAGAAGAGTTAGACACTTTGCCTAGGATCCACTGTGCGC

DDBJ/EMBL/GenBank (accession number: LC123593)
>MCg2_S/LC123593.seq
CGCACCGGGGATCCTAGGCGATTCTTGATCGCGCTTATTAGCTAACCAATTTAATTTGGT
GTTGAAGTGTTGACACGCTCTCTAACACATGGGGCAGCTTATCAGCTTCTTTCAGGAGAT
TCCTGTTTTTCTACAGGAAGCTCTGAACATCGCTTTAGTGGCTGTTAGTCTCATAGCTGT
CATCAAAGGCATCATTAACCTTTACAAAAGTGGTCTCTTCCAGTTCATCTTCTTTCTCCT
CCTAGCAGGGAGGTCCTGCTCGGAAGAAGCTTTCAAAATCGGACTGCACACTGAGTTCCA
GACTGTGTCCTTCTCAATGGTGGGTCTCTTTTCCAACAATCCACATGACCTACCTTTGTT
GTGTACCTTAAACAAGAGCCATCTTTACATTAAGGGGGGCAATGCTTCATTTCAGATCAG
CTTTGATGATATTGCAGTATTGTTGCCACAGTATGATGTTATAATACAACATCCAGCAGA
TATGAGCTGGTGTTCCAAAAGTGATGATCAAATTTGGTTGTCTCAGTGGTTCATGAATGC
TGTGGGACATGATTGGCATCTAGACCCACCATTTCTGTGTAGGAACCGTGCAAAGACAGA
AGGCTTCATCTTTCAAGTCAACACCTCCAAGACTGGTGTCAATGGAAATTATGCTAAGAA
GTTTAAGACTGGCATGCATCATTTATATAGAGAATATCCTGACCCTTGCTTGAATGGCAA
ACTGTGCTTAATGAAGGCACAACCTACCAGTTGGCCTCTCCAATGTCCACTCGACCACGT
TAACACATTACACTTCCTTACAAGAGGTAAAAACATTCAACTTCCAAGGAGGTCCTTGAA
AGCATTCTTCTCCTGGTCTTTGACAGACTCATCCGGCAAGGATACCCCTGGAGGCTATTG
TCTAGAAGAGTGGATGCTCGTAGCAGCCAAAATGAAGTGTTTTGGCAATACTGCTGTAGC
AAAATGCAATTTGAATCATGACTCTGAATTCTGTGACATGTTGAGGCTCTTTGATTACAA
CAAAAATGCTATCAAAACCCTAAATGATGAAACTAAGAAACAAGTAAATCTGATGGGGCA
GACAATCAATGCCCTGATATCTGACAATTTATTGATGAAAAACAAAATTAGGGAACTGAT
GAGTGTCCCTTACTGCAATTACACAAAATTTTGGTATGTCAACCACACACTTTCAGGACA
ACACTCATTACCAAGGTGCTGGTTAATAAAAAACAACAGCTATTTGAACATCTCTGACTT
CCGTAATGACTGGATATTAGAAAGTGACTTCTTAATTTCTGAAATGCTAAGCAAAGAGTA
TTCGGACAGGCAGGGTAAAACTCCTTTGACTTTAGTTGACATTTGTATCTGGAGCACAAT
TTTCTTCACAGCATCATTGTTCCTTCATCTAGTTGGA-ATACCCACCCATCGACACCTCA
AAGGCGAAGCCTGTCCTTTGCCTCATAAGCTGGACAGCTTCGGAGGTTGTAGATGTGGCA
AATATCCCAGATTGAAGAAACCCACCATCTGGCACAAAAGACATTAAACACAGCCAAGAC
CCCTGCCGACCCGGGCCCAGCCCGGGTCGGCGGGGCCCCCCAGTCCGCGGCTCTGCCGCG
GACTGGGGAGGCACTGCTTACAGTGCAAAGGCTGCCTTGGGTAGAAATAGAAGGCTGAGA
GGTAAAAGTGCCACTGGTTCTTCATCTACCATTTTGCCATCCATCACTGATTGATACATG
ATACAATCCAACAGAGCACAGTGAGGTTCACCAGAACTCAATTTGAATGGAGAGTCTTTT
TCTCTCTTTTTCTTGTTGACAACAACTCCATTATGATGTTTGCAAAGATGTCCAAACTTC
TCCCAAATTTGCTGATCATAGAGTCTTGCTTGATCAGATGTGAGTTTCACATCTACCAGC
TTTAAATCCTTCCGTCCATGAATGTCTAAAAGTTTCCTTATGTCGTCAGAACCTTGAGTT
GTAATCACCATATCTTGTGGTAGTAAACCTATTACATAACTTAATACTCCTGGCATTGCA
TCCTCGATGTCTTGCATCAAGATGCCATGTGAATGTCTGCTGCCATTTTTGAAGCCCTTC
TCATCATGTGGCTTTCTAAAACAATGAATATAATGCTTGTTGGCTGGCTGATATAGTGCC
AATTCCACAGGGTCTGTTGGAGGCCCTTCAATGTCAAGCCACAGTGTATTAGTGGGGTCT
AACTTCTTTACAGCCTCTTTAATCACTGCTTCTTGCATTTCAGTCAGGTTTGCTAGTCTT
AGATTCTGACCGTTTTTCTCGGGTTGTCTCGGTCCAACTTGAGGTTTCTTTGTTAGATCA
ACACTTGTGTTGTCCCAAGATCTCCCAAGAATTTGTGATCTGGAACCAATGTAAGGCCAC
CCGTCACCTGAGAGACACAATTTATACAGAAGGTTCTCATAAGGATTTCGATTCCCAGGT
TTCTCGTCTATAAACATCCCCTCTTTTCGCTTGACTTGCAGAGTTGATTTAATCAAGCTG
GAGAAATTATCAGGAGTGACCCTAATTGTTTCCAGCATGTTCCCACCGTCAAGTATTGAG
GCGCCAGCTTTCACAGCAGCTGACAGACTGAAGTTATAGCCAGAGATGTTGATGGAGCTC
TCGTCCTTAGTTACGATCTGCAAGCATTCGTGCTCTAGAGTTAGCTTGTCAAGGTCATTT
AAATTCGGATATTTAACAGTGTATAACAGGCCCAGAGATGTCAACGCTTGGACCACACTA
TTCATCGTTTCTCCACCCTGGACAGTCATACAAGCGATTGTGAGAGCTGGCATGGAACCA
AACTGATTATTCAGTTTTGATGGATCTGAGACATCCCAGATTCTAACCACACCATTTCCA
GCACCTCTCTGCTGTTGGAATCCCAGGGTCTTCAAGATTTCTGACCTCTTTGTCAGTTGC
AGCTGTGACAAATTACCCATGTACAGCCCTTGGGGACCTGACTCAGTCCTCTTTATCTTG
TTCTTCAGTTTTTCCAAGTCTGATGCAAGCTCCATCAATTCATCTCTCCCCAGATCTCCA
ATCTTGAATATGGTGTTTTTCTGGATGCTTTTCATAGACATGAGCCTATCCACTTCTTTG
TTTAAATCCCTCAGCTTGTTCAGATCCTCTTCACCCCTTTTGTTCTTTCTGAGAGCCCTC
TGCACTTGTGAAACCTGGTTGAAGTCGATGCTGTCAGCTATGAGCTTGGCATCTTTCAAC

ACATCTGTTTTCACAGTGTGGGTGAACTGACTCAGGCCTCTTCTCAGTGACTGAGTCCAC
CGAAAGCTGGGAATTTCCTTGGAGTGGGCCATAGTGGCTAATAACCAAAGGATTTGAAAT
AAAGCTCAGTAGAAGAGTTAGACACTTTGCCTAGGATCCACTGTGCGC

DDBJ/EMBL/GenBank (accession number: LC123594)
>MCg3_S/LC123594.seq
CGCACCGGGGATCCTAGGCGATTCTTGATCGCGCTTATTAGCTAACCAATTTAATTTGGT
GTTGAAGTGTTGACACGCTCTCTAACACATGGGGCAGCTTATCAGCTTCTTTCAGGAGAT
TCCTGTTTTTCTACAGGAAGCTCTGAACATCGCTTTAGTGGCTGTTAGTCTCATAGCTGT
CATCAAAGGCATCATTAACCTTTACAAAAGTGGTCTCTTCCAGTTCATCTTCTTTCTCCT
CCTAGCAGGGAGGTCCTGCTCGGAAGAAGCTTTCAAAATCGGACTGCACACTGAGTTCCA
GACTGTGTCCTTCTCAATGGTGGGTCTCTTTTCCAACAATCCACATGACCTACCTTTGTT
GTGTACCTTAAACAAGAGCCATCTTTACATTAAGGGGGGCAATGCTTCATTTCAGATCAG
CTTTGATGATATTGCAGTATTGTTGCCACAGTATGATGTTATAATACAACATCCAGCAGA
TATGAGCTGGTGTTCCAAAAGTGATGATCAAATTTGGTTGTCTCAGTGGTTCATGAATGC
TGTGGGACATGATTGGCATCTAGACCCACCATTTCTGTGTAGGAACCGTGCAAAGACAGA
AGGCTTCATCTTTCAAGTCAACACCTCCAAGACTGGTGTCAATGGAAATTATGCTAAGAA
GTTTAAGACTGGCATGCATCATTTATATAGAGAATATCCTGACCCTTGCTTGAATGGCAA
ACTGTGCTTAATGAAGGCACAACCTACCAGTTGGCCTCTCCAATGTCCACTCGACCACGT
TAACACATTACACTTCCTTACAAGAGGTAAAAACATTCAACTTCCAAGGAGGTCCTTGAA
AGCATTCTTCTCCTGGTCTTTGACAGACTCATCCGGCAAGGATACCCCTGGAGGCTATTG
TCTAGAAGAGTGGATGCTCGTAGCAGCCAAAATGAAGTGTTTTGGCAATACTGCTGTAGC
AAAATGCAATTTGAATCATGACTCTGAATTCTGTGACATGTTGAGGCTCTTTGATTACAA
CAAAAATGCTATCAAAACCCTAAATGATGAAACTAAGAAACAAGTAAATCTGATGGGGCA
GACAATCAATGCCCTGATATCTGACAATTTATTGATGAAAAACAAAATTAGGGAACTGAT
GAGTGTCCCTTACTGCAATTACACAAAATTTTGGTATGTCAACCACACACTTTCAGGACA
ACACTCATTACCAAGGTGCTGGTTAATAAAAAACAACAGCTATTTGAACATCTCTGACTT
CCGTAATGACTGGATATTAGAAAGTGACTTCTTAATTTCTGAAATGCTAAGCAAAGAGTA
TTCGGACAGGCAGGGTAAAACTCCTTTGACTTTAGTTGACATCTGTATTTGGAGCACAGT
ATTCTTCACAGCGTCACTCTTCCTTCA-CTTGGTGGGTATACCCACCCATCGACACCTCA
AAGGCGAAGCCTGTCCTTTGCCTCATAAGCTGGACAGCTTCGGAGGTTGTAGATGTGGCA
AATATCCCAGATTGAAGAAACCCACCATCTGGCACAAAAGACATTAAACACAGCCAAGAC
CCCTGCCGACCCGGGCCCAGCCCGGGTCGGCGGGGCCCCCCAGTCCGCGGCTCTGCCGCG
GACTGGGGAGGCACTGCTTACAGTGCAAAGGCTGCCTTGGGTAGAAATAGAAGGCTGAGA
GGTAAAAGTGCCACTGGTTCTTCATCTACCATTTTGCCATCCATCACTGATTGATACATG
ATACAATCCAACAGAGCACAGTGAGGTTCACCAGAACTCAATTTGAATGGAGAGTCTTTT
TCTCTCTTTTTCTTGTTGACAACAACTCCATTATGATGTTTGCAAAGATGTCCAAACTTC
TCCCAAATTTGCTGATCATAGAGTCTTGCTTGATCAGATGTGAGTTTCACATCTACCAGC
TTTAAATCCTTCCGTCCATGAATGTCTAAAAGTTTCCTTATGTCGTCAGAACCTTGAGTT
GTAATCACCATATCTTGTGGTAGTAAACCTATTACATAACTTAATACTCCTGGCATTGCA
TCCTCGATGTCTTGCATCAAGATGCCATGTGAATGTCTGCTGCCATTTTTGAAGCCCTTC
TCATCATGTGGCTTTCTAAAACAATGAATATAATGCTTGTTGGCTGGCTGATATAGTGCC
AATTCCACAGGGTCTGTTGGAGGCCCTTCAATGTCAAGCCACAGTGTATTAGTGGGGTCT
AACTTCTTTACAGCCTCTTTAATCACTGCTTCTTGCATTTCAGTCAGGTTTGCTAGTCTT
AGATTCTGACCGTTTTTCTCGGGTTGTCTCGGTCCAACTTGAGGTTTCTTTGTTAGATCA
ACACTTGTGTTGTCCCAAGATCTCCCAAGAATTTGTGATCTGGAACCAATGTAAGGCCAC
CCGTCACCTGAGAGACACAATTTATACAGAAGGTTCTCATAAGGATTTCGATTCCCAGGT
TTCTCGTCTATAAACATCCCCTCTTTTCGCTTGACTTGCAGAGTTGATTTAATCAAGCTG
GAGAAATTATCAGGAGTGACCCTAATTGTTTCCAGCATGTTCCCACCGTCAAGTATTGAG
GCGCCAGCTTTCACAGCAGCTGACAGACTGAAGTTATAGCCAGAGATGTTGATGGAGCTC
TCGTCCTTAGTTACGATCTGCAAGCATTCGTGCTCTAGAGTTAGCTTGTCAAGGTCATTT
AAATTCGGATATTTAACAGTGTATAACAGGCCCAGAGATGTCAACGCTTGGACCACACTA
TTCATCGTTTCTCCACCCTGGACAGTCATACAAGCGATTGTGAGAGCTGGCATGGAACCA
AACTGATTATTCAGTTTTGATGGATCTGAGACATCCCAGATTCTAACCACACCATTTCCA
GCACCTCTCTGCTGTTGGAATCCCAGGGTCTTCAAGATTTCTGACCTCTTTGTCAGTTGC
AGCTGTGACAAATTACCCATGTACAGCCCTTGGGGACCTGACTCAGTCCTCTTTATCTTG
TTCTTCAGTTTTTCCAAGTCTGATGCAAGCTCCATCAATTCATCTCTCCCCAGATCTCCA
ATCTTGAATATGGTGTTTTTCTGGATGCTTTTCATAGACATGAGCCTATCCACTTCTTTG

TTTAAATCCCTCAGCTTGTTCAGATCCTCTTCACCCCTTTTGTTCTTTCTGAGAGCCCTC
TGCACTTGTGAAACCTGGTTGAAGTCGATGCTGTCAGCTATGAGCTTGGCATCTTTCAAC
ACATCTGTTTTCACAGTGTGGGTGAACTGACTCAGGCCTCTTCTCAGTGACTGAGTCCAC
CGAAAGCTGGGAATTTCCTTGGAGTGAGCCATAGTGGCTAATAACCAAAGGATTTGAAAT
AAAGCTCAGTAGAAGAGTTAGACACTTTGCCTAGGATCCACTGTGCGC
